# The Effects of Spaceflight Factors on the Human Plasma Proteome, Including Both Real Space Missions and Ground-Based Experiments

**DOI:** 10.3390/ijms20133194

**Published:** 2019-06-29

**Authors:** Alexander G. Brzhozovskiy, Alexey S. Kononikhin, Lyudmila Ch. Pastushkova, Daria N. Kashirina, Maria I. Indeykina, Igor A. Popov, Marc-Antoine Custaud, Irina M. Larina, Evgeny N. Nikolaev

**Affiliations:** 1 Institute of Biomedical Problems, Russian Federation State Scientific Research Center, Russian Academy of Sciences, 119991 Moscow, Russia; 2 Laboratory of mass spectrometry, CDISE, Skolkovo Institute of Science and Technology, 121205 Moscow, Russia; 3 V.L. Talrose Institute for Energy Problems of Chemical Physics, N.N. Semenov Federal Center of Chemical Physic of RAS, 119334 Moscow, Russia; 4 Emanuel Institute of Biochemical Physics, Russian Academy of Sciences, 119334 Moscow, Russia; 5 Laboratory of Ion and Molecular Physics, Moscow Institute of Physics and Technology, Dolgoprudny, 141701 Moscow, Russia; 6 MitoVasc laboratory, Angers University, 49035 Angers, France

**Keywords:** spaceflight, ground-based experiments, mass-spectrometry, astronauts, head-down bed rest, dry immersion, proteomics, extreme conditions

## Abstract

The aim of the study was to compare proteomic data on the effects of spaceflight factors on the human body, including both real space missions and ground-based experiments. LC–MS/MS-based proteomic analysis of blood plasma samples obtained from 13 cosmonauts before and after long-duration (169–199 days) missions on the International Space Station (ISS) and for five healthy men included in 21-day-long head-down bed rest (HDBR) and dry immersion experiments were performed. The semi-quantitative label-free analysis revealed significantly changed proteins: 19 proteins were significantly different on the first (+1) day after landing with respect to background levels; 44 proteins significantly changed during HDBR and 31 changed in the dry immersion experiment. Comparative analysis revealed nine common proteins (*A1BG, A2M, SERPINA1, SERPINA3, SERPING1, SERPINC1, HP, CFB, TF*), which changed their levels after landing, as well as in both ground-based experiments. Common processes, such as platelet degranulation, hemostasis, post-translational protein phosphorylation and processes of protein metabolism, indicate common pathogenesis in ground experiments and during spaceflight. Dissimilarity in the lists of significantly changed proteins could be explained by the differences in the dynamics of effective development in the ground-based experiments. Data are available via ProteomeXchange using the identifier PXD013305.

## 1. Introduction

Today, world space agencies are faced with several tasks, including equipping the International Space Station (ISS) with new modules for fundamental space research aboard the station, landing on the moon and further colonization of the lunar surface. These tasks require long-duration spaceflights (SF) for over a year onboard manned space objects. During the entire flight, a complex of extreme factors, such as microgravity, acceleration and cosmic radiation affects the cosmonaut’s body [1,2].

To determine what changes occur under the influence of spaceflight factors, experiments on cell cultures and experiments on laboratory animals were conducted [3,4,5]. During the flight, a decrease in the levels of myofibrillar and sarcoplasmic proteins was observed. Activation of proteolysis of cytoskeletal proteins, such as desmin and titin, underlies muscular atrophy, which causes a decrease in the contractile properties of muscles, endurance, and physical performance [2,5]. In addition, changes in the cardiovascular system were detected under the influence of spaceflight factors [6]. An increase in the level of calcium ions and the parathyroid hormone and a decrease in the level of calcitonin were observed on the first day after landing [7]. One of the most accessible methods for studying the effects of spaceflight on the human body is ground-based experiments on healthy volunteers. However, there are certain ethical restrictions that allow only experiments with physical inactivity and diet modification, in particular, modification of the composition of basic nutrients, as well as exercise tests. Some experiments were specifically designed to simulate physiological changes under the influence of separate factors of spaceflight such as head-down bed rest, dry immersion, and isolation in sealed chambers [8,9]. Head down bed rest (HDBR) is one of the experimental models that limits the mobility of a person to strict bed rest, while the head of the bed is lowered relative to the horizontal axis [8]. During these experiments, loss of muscle mass occurs due to a decrease in protein synthesis [10], while no increase in the rate of proteolysis of myofibrils nor activation of the ubiquitin-proteasome pathway of protein degradation is observed [11,12]. This experiment helps to investigate the functional adaptation of muscles to the decrease in motor activity, specifically for spaceflight and bed rest [13]. Dry immersion is another ground-based experiment that is widely used in gravitational physiology to simulate the early effects of microgravity. During the experiment, volunteers are immersed in thermoneutral water covered with a special elastic waterproof fabric. It is known that this model accurately reproduces the cardiovascular, motor, and other changes observed during spaceflight [9].

Nowadays “omics” methods, in particular, mass spectrometry, allow the identification of adaptive changes that occur in the body under the influence of spaceflight factors. Previously, such studies were carried out using targeted methods (mainly, enzyme immunoassays of specific proteins) [3,14], but they significantly limit the number of studied proteins. Untargeted mass-spectrometry-based proteomics approach allows us to investigate the significantly larger number of proteins in a sample (the dynamic range of protein concentrations in plasma covers up to 10–11 orders of magnitude) [15] and to find new biomarkers of various extreme states.

Proteomic studies of spaceflight effects are mostly limited to various models, such as cultured cells [3,11,12] and ground-based experiments [16,17,18,19] because nowadays none of the current “omics” technologies could be applied during spaceflight. Moriggi and colleagues used 2-D electrophoresis in combination with matrix assisted laser desorption/ionization (MALDI) mass spectrometry and high-performance liquid chromatography with tandem mass spectrometry to analyze muscle biopsies after 55 days of horizontal bed rest [20]. The authors observed a significant decrease in the activity of proteins involved in aerobic metabolism in comparison to the control group. Potential biomarker proteins can be detected in various biological fluids such as blood or urine. A quantitative proteomic study based on selected reaction monitoring with stable isotope-labeled (SIS) peptide standards for 84 proteins encoded by the human chromosome 18 was performed with the aim to provide a reference for future studies of human plasma chromosome 18 proteome changes during spaceflight [17]. Investigation of protein abundance variability in 56 urine samples, collected from six volunteers participating in the MARS-500 experiment was conducted using a targeted parallel reaction monitoring (PRM) method [18]. However, the complete set of factors that affected the human body onboard the ISS is impossible to be simulated in ground-based settings. Only a few works were conducted using biological samples (blood, urine, hair, saliva, and muscle biopsy material) obtained in real spaceflight conditions [21,22,23,24].

The aim of the study was to compare proteomic data on the effects of spaceflight factors on the human body, including both real space missions and ground-based experiments. To identify protein changes under the influence of spaceflight factors, semi-quantitative label-free proteomic analysis of blood plasma samples obtained from 13 cosmonauts before and after long-duration (169–199 days) missions on the ISS (International Space Station) was performed. In order to determine the common processes and pathways that are involved in adaption to spaceflight factors, proteomic analysis of blood plasma samples collected during ground-based experiments (HDBR and dry immersion) was also carried out.

## 2. Results and Discussion

### 2.1. Proteomic Analysis of Blood Plasma in Ground-Based Experiments and Spaceflight

#### 2.1.1. Head-Down Bed Rest (HDBR)

**Figure 1:**
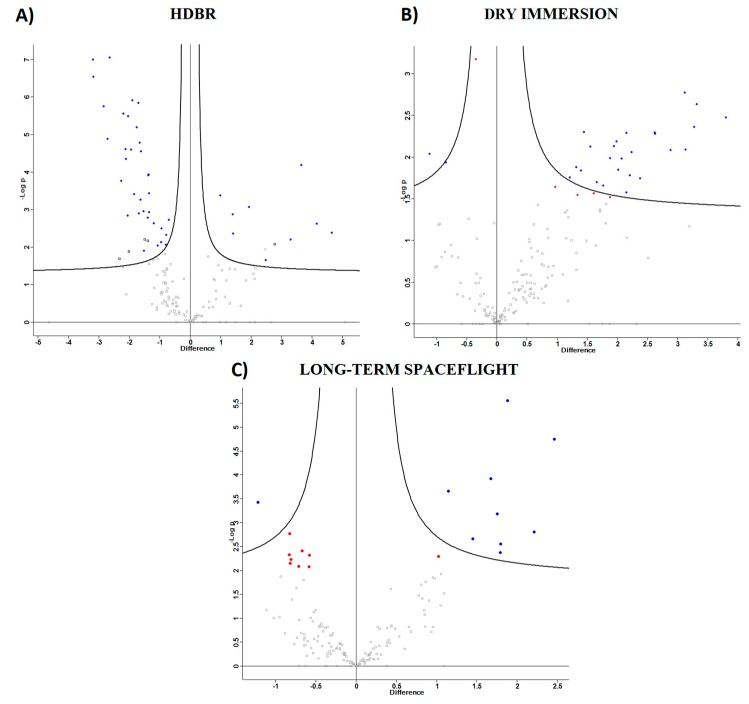
A volcano plot representing the results of the *t*-tests (*p*-value > 0.01). The difference for each protein was plotted against the –log10 of the *p*-value. (**A**) Proteins are significantly different from the background during head-down bed rest (HDBR). (**B**) Proteins significantly differing from the background in the dry immersion experiment. (**C**) Proteins significantly changed at +1 days after landing with respect to their background levels. Colours indicates volcano plot *t*-tests significance with a permutation-based false discovery rate (FDR) calculation for significantly changed proteins: blue—FDR < 0.05; red—FDR > 0.05.

Semi-quantitative label-free proteomic analysis of 10 samples collected from five volunteers participating in ground-based HDBR was performed. Plasma samples were collected a day before the experiment (background) and on the 21st day of the experiment. The mass-spectrometry analysis resulted in 284 detected proteins. Welch’s *t*-tests (*p*-value > 0.01) revealed 44 significantly changed proteins on the 21st day of the experiment, among them, the levels of 10 proteins had decreased (*MASP2, QSOX1, IGKC, IGHG4, APOC2, ACTN1, PLTP, TLN1* Histone *H2A*, Actin) and 34 had increased (*ATRN, CFB, SERPINC1, SERPINA1, SERPINA3, AGT, A2M, C5*, and others) (Figure 1A, Table 1).

Gene Ontology (GO) annotations were used to characterize these proteins by their localization in cellular components and molecular function via the PANTHER system. Most proteins with changed expression levels under the influence of HDBR conditions function in binding (40%), catalytic activity (transferase, oxidoreductase, etc.) (30%) and in the regulation of molecular functions (26%). GO annotation via Cellular compound database revealed that these proteins are localized in the cell (15.6%), extracellular region (56.3%), membrane (9.4%), organelles (12.5%), or are part of protein-containing complexes (6.3%).

#### 2.1.2. Dry Immersion

The mass-spectrometry analysis of 10 samples collected from five volunteers participating in a ground-based dry immersion experiment resulted in 274 detected proteins. Plasma samples were collected a day before the experiment (background) and on the 21st day of the experiment. The label-free proteomic analysis resulted in 31 significantly changed proteins. Almost all significantly changed proteins increase their background values after 21 days of the experiment (Figure 1B, Table 2).

The vast majority of significantly changed proteins under the conditions of “dry” immersion perform their function in the cell (7.4%), extracellular region (51.9%), membrane (11.1%), organelles (7.4%), or are part of protein-containing complexes (22.2%). The main functions of these proteins are binding (37.5%), catalytic activity (transferase, oxidoreductase, etc.) (33%), regulators of molecular functions (21%), signaling (4%), as well as structural proteins.

### 2.2. Spaceflight

**Figure 2:**
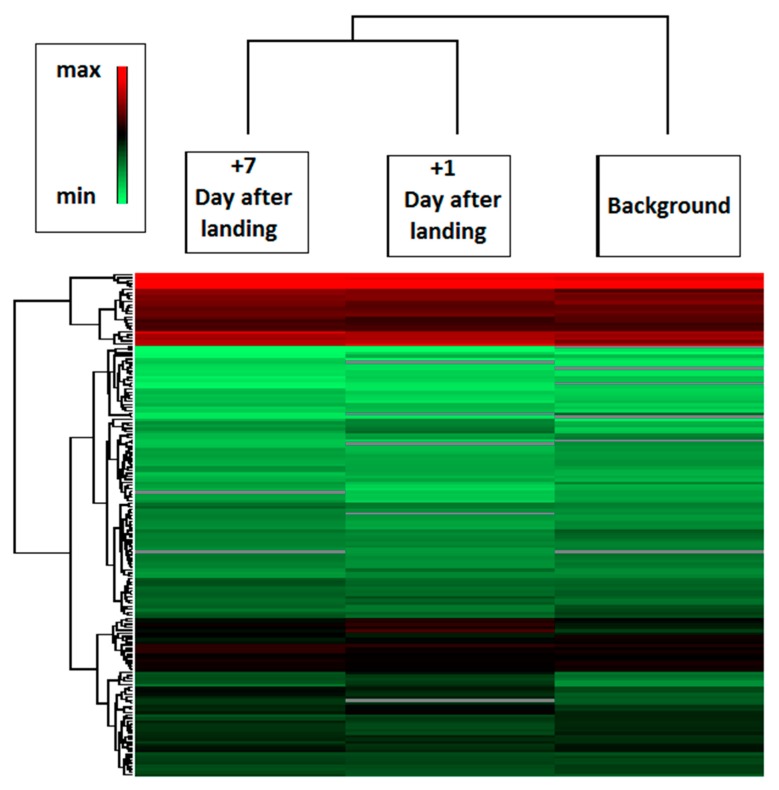
The heat map analysis represents the hierarchical clustering of samples set before and after the spaceflight. Hierarchical clustering of proteomic composition was performed using logarithmized label-free quantification (LFQ) intensities. The strength of the colors indicates the relative abundance of the protein in different groups.

Semi-quantitative label-free proteomic analysis of 39 samples collected from 13 Russian cosmonauts before and after long-term (169–199 days) spaceflights were performed. Plasma samples were collected at three different time periods: background (six months before flight), on the first (+1 day) and seventh (+7 days) days after landing. The mass-spectrometry analysis resulted in the identification of 419 different proteins. Evaluation of the proteomic composition in different groups was performed via hierarchical clustering based on the average levels of label-free quantification (LFQ) intensities (Figure 2). Comparison of the results shows that samples obtained +1 and +7 days after landing are more similar with respect to their background levels. Apparently, shifts of proteomic composition conducted under the influence of spaceflight factors (including adaptation to terrestrial conditions) and do not fully restore its preflight values even after +7 days. Statistical analysis revealed 19 proteins, which were significantly changed (presumably under the influence of spaceflight factors) at +1 day with respect to its background level. Most of these proteins did not restore their preflight levels to that of day +7 (Table 1). A total of 10 proteins increased their levels (*HP, CFB, SERPINC1, SERPINA1, SERPINA3, A2M, TF, A1BG, SERPING1, SAA1*) and nine decreased their levels (*QSOX1, F13A1, F13B, EFEMP1, F5, CDH1, FLNA, CDH5, PON3*), as compared to preflight values (Figure 1C, Table 3).

Enrichment analysis was conducted via the GO database (pathways and processes) using the STRING and Panther GO software. Proteins significantly changed +1 day after long-term spaceflight perform the following functions: binding (37.5%), catalytic activity (transferase, oxidoreductase, etc.) (33%), regulation of molecular functions (21%), transduction (signaling) (4%), as well as structural function. These proteins were localized in cell junctions (6%), cell (19%), extracellular region (44%), membrane (12.5%), organelles (12.5%), or were part of protein-contained complexes (6%).

### 2.3. Comparison of Ground-Based Experiments and Spaceflight

**Figure 3:**
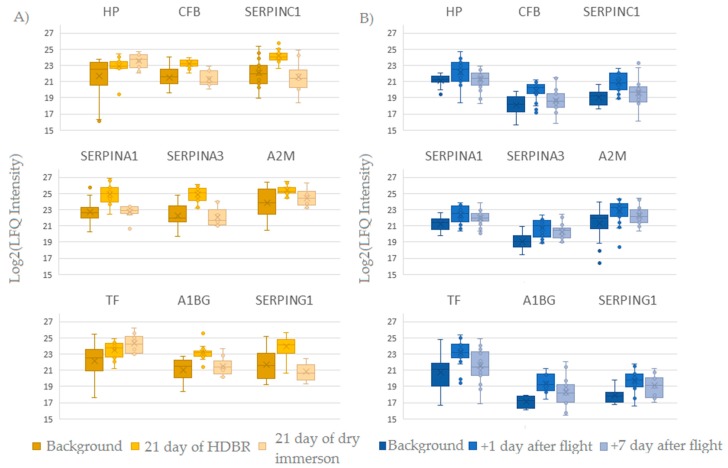
The label-free quantification (LFQ) intensity box plot for nine proteins that change their level at the 21st day in the ground-based experiments (**A**) and after space flight (**B**). The LFQ values are plotted in Log2(x) scale along the vertical axis.

During spaceflight, several extreme factors affect the human body, such as microgravity [21], the shift of liquid media [22] and stress [23] that trigger adaptation processes of the organism. Then, at the final stage of the flight, during landing, the cosmonaut’s body undergoes stress caused by overloads. After landing, adaptation to Earth conditions commences. To identify the key biological processes that are involved in adaptive changes under the influence of spaceflight factors, analysis of the proteomic composition was conducted. To determine the effect of certain factors on proteome changes during spaceflight, samples collected during ground-based experiments—HDBR and “dry” immersion—were analyzed. The statistical label-free analysis revealed nine proteins that change their level at +1 day after landing as well as in the ground-based experiments (Figure 3). Presumably, these proteins change their level under the influence of major spaceflight factors such as gravitational changes and fluid redistribution. The revealed changes are most pronounced on the 21st day of the HDBR experiment. Most of these nine proteins are involved in the processes of platelet degranulation (6), proteolysis regulation (6), immune response (7) and response to external stimuli (9) or stress (7). Significant changes of Serotransferrin levels during the space flight and ground-based experiments may be associated with of space flight anemia—reduction in circulating red blood cells and the decrement of plasma volume in a 10% to 15% as an adaptation to weightlessness followed by an increase of iron during long-duration space flight [24].

**Figure 4:**
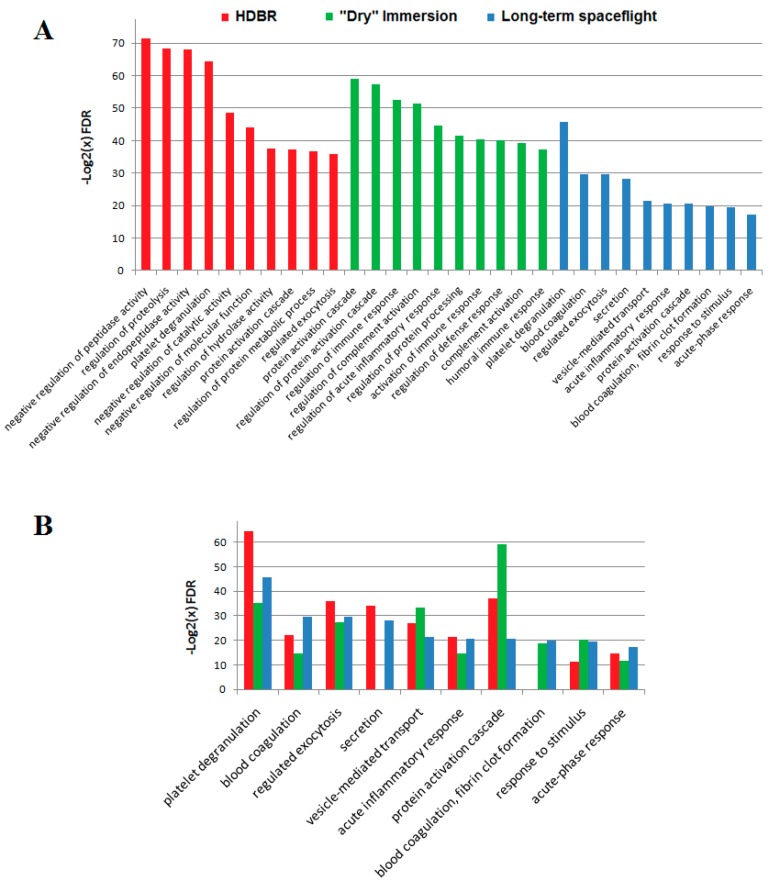
The histogram of gene ontology (GO) term enrichment in ground-based experiments and during spaceflight. (**A**) The list of the top 10 processes in which significantly changed proteins take part. (**B**) Comparison of the top 10 processes enriched on +1 days after landing with similar processes in HDBR and Dry immersion. The ordinate represents the enriched GO terms, and the abscissa represents the −log2(FDR).

In the results of the STRING analysis using GO databases (processes, pathways), a list of the top 10 processes was identified (Figure 4A). During the spaceflight, the most significant was the participation of *A1BG, A2M, F13A1, F5, FLNA, QSOX1, SERPINA1, SERPINA3, SERPING1, TF* proteins in the processes of platelet degranulation at the sites of vascular damage. It is known that during platelet activation, various proteins (including *A2M, F13A1, F13B, F5, SERPINA1, SERPINA3, SERPINC1*, and *SERPING1*) are released, mainly from alpha-granules and lysosomes, as well as from lysed cells. They perform autocrine or paracrine functions by modulating cell signaling [25]. The participation of proteins in blood coagulation processes, namely, in the formation of fibrin fibers through the classical (*F5, F13A1, F13B, SERPINC1*) and internal pathways (*A2M, SERPING1, SERPINC1*) are also significant. Activation of these systems was also observed during ground-based experiments. However, the activation of these systems does not appear to be connected to vascular wall injuries but with the maintenance of homeostasis in response to extreme conditions. This is confirmed by the participation of significantly changed proteins in the regulation of the cytosolic Ca2+ level (*A1BG, A2M, F13A1, F5, FLNA, QSOX1, SERPINA1, SERPINA3, SERPING1, TF*) [26]. A number of authors observed the immune system dysregulation during long-duration spaceflights [27]. Significant weakening of the lymphocyte response to mitogenic stimulation was observed [28], as well as changes in wound-healing processes and in the function of granulocytes [1,29]. In this study, the proteomic analysis allowed for the determination of changes in the levels of regulatory proteins, including members of the coagulation cascades (*F5, F13A1, F13B*, etc.) and immune-response proteins (*C3, CFB, SPP1, C4A, ORM1, ORM2, CD14*). Among the significantly changing proteins associated with the immune response, most proteins are important participants of the innate immune system. Potentially, these proteins can increase their level in the course of adaptation to the Earth’s microbiota.

During ground-based experiments, the participation of significantly changed proteins in platelet degranulation processes was also observed, mainly in the HDBR experiment (*A1BG, A2M, ACTN1, ECM1, HRG, ITIH3, ITIH4, KNG1, QSOX1, SERPINA1, SERPINA4, SERPINF2, SERPING1, TLN1*). The participation of proteins in the coagulation processes was less enhanced and, especially, in the process of fibrin fiber formation in both ground experiments. During the dry immersion experiment, the effect on proteins participating in the remodeling of the extracellular matrix was more pronounced (*FGA, FGB, FGG, TTR, VTN*). On the contrary, during HDBR signaling by Rho GTPases, *A2M, ACTB, ACTG1, HIST1*, and *H2AJ* were observed, as well as during long-term spaceflight (*A2M, CDH1, FLNA*). Several proteins (*C3, TTR and APOB*) changed their levels in both ground-based experiments. Besides its role in coagulation, *C3* prolongs oxidative stress [30].

Common processes (Figure 4B) indicate common pathogenesis in ground-based experiments and during spaceflight. Any dissimilarity in the lists of significantly changed proteins could be explained by the differences in the dynamics of the development of effects in the ground-based experiments. The changes occurring during the dry immersion experiment develop rather quickly [9]—faster than during HDBR. Therefore, on the 21st day of the experiments, the physiological systems of the organism are in different states. Additionally, differences in the degeneration of some processes in HDBR may be due to the increased influence of the liquid media redistribution.

GO terms enrichment analysis of flight data indicates that even after 6 months of spaceflight, the mechanism of vascular platelet hemostasis remains activated. Platelet degranulation is the final stage of fibrin clot formation in response to injuries of the vascular wall. Previously some experiments show that petechiasis can be developed under the influence of accelerations between 5 G and 9 G (for example, during centrifugation or descent) in those parts of the human body where pressure load is highest [31]. Some evidence shows that petechiasis can be formed after bed rest studies [32]. Such changes could be associated with endothelium and blood vessel integrity. Spaceflight modifies the functions of endothelial cells which also contributes to the changes in hemostasis [33]. We suppose that observed changes demonstrate physiological adaptation to spaceflight conditions. This is supported by the fact that the changes do not recover up to +7 days after spaceflight.

## 3. Materials and Methods

### 3.1. Sample Collection

Blood plasma samples were collected from 13 cosmonauts (male, age: 46 ± 6 years) before and after long-duration (169–199 days) missions on the ISS (International Space Station). All subjects provided written informed consent to participate in the ‘‘Proteome’’ experiment.

Five healthy men from 20 to 44 years of age were included in the head-down bedrest experiment (HDBR). They voluntarily remained in a bed rest position with an angle of inclination of the longitudinal axis of the body relative to the horizontal position of 6° for 21 days. Subjects characteristics were as follows: 34.3 ± 8.3 year (age), 1.76 ± 0.06 m (height), 69.8 ± 8.0 kg (weight), body mass index 22.4 ± 1.7 (BMI). All the subjects consumed controlled amount of all nutrients in the diet such as proteins, fats, carbohydrates and vitamins, calculated (calories per day) according to general recommendations by the European Space Agency (ESA). The studies were conducted under controlled life conditions of the volunteers at the MEDES research center in Toulouse, France. The examined group was not exposed to any additional stress to prevent the development of adaptive changes in physiological systems. More detailed subjects’ characteristics, medical check-up, inclusion and exclusion criteria, as well as dropout criteria and study design have been described previously [34].

Samples from the dry immersion study were obtained from 5 volunteers participating in a 21-day-long experiment. Subject characteristics were as follows: 27.5 ± 3.7 year (age), 1.76 ± 0.05 m (height), 75.7 ± 8.4 kg (weight), 24.1 ± 1.3 (BMI). All the subjects consumed a controlled amount of all nutrients in the diet such as proteins, fats, carbohydrates and vitamins, calculated (calories per day) according to general recommendations by the World Health Organization (WHO). This study was conducted under controlled life conditions at the Institute of Biomedical Problems (IBP) of the Russian Federation State Scientific.

During ground-based experiments, cushion using was not restricted. Participants selected for the experiments were non-smokers, non-drugs takers and did not exhibit acute or chronic pathologies which could affect the physiological data. The volunteers had normal clinical and paramedical examination and laboratory tests (hematology and blood chemistry). All the participants gave their informed consent to the experimental conditions after the details of the protocol were explained to them.

All blood samples (~6 mL) were taken from a vein in the cubital fossa, the collection was done in commercial Monovette tubes (SARSTEDT, Germany) containing EDTA (K3) as the anticoagulant. No protease inhibitors or antimicrobial agents were added. The samples were centrifuged for plasma separation (2000 rpm for 15 min, +4 °C) immediately after collection. The supernatant was frozen at −80 °C and stored before further sample preparation for LC-MS analysis. All methods were performed in accordance with the relevant guidelines and regulations.

### 3.2. LC-MS/MS Proteomic Analysis

For proteomic analysis, 200 μL of blood plasma was used for high-abundance protein depletion (ProteoMiner^TM^, BioRad, Hercules, CA, USA) and a concentration of low-abundance proteins. The samples were prepared via the filter-aided sample preparation (FASP) [35] protocol using 10 kDa filters (Merc, London, UK). Plasma proteins were reduced using 0.1 mol/L dithiothreitol (DTT) in 8 mol/L Urea (pH 8.5); alkylated with 0.55 mol/L iodoacetamide and digested using trypsin (17 h, 37 °C).

The tryptic peptide fraction (injection volume 2 µL) was analyzed in triplicate on a nano-HPLC Dionex Ultimate3000 system (Thermo Fisher Scientific, Waltham, MA, USA) coupled to a MaXis 4G (Bruker Daltonics, Bremen, Germany) using a nanospray ion source (positive ion mode, 1600 V) (Bruker Daltonics). HPLC separation was performed on a C18 capillary column (75 µm × 50 cm, C18, 3 µm, 100 A) (Thermo Fisher Scientific) at a flow rate of 0.3 µL/min by gradient elution. The mobile phase A was 0.1% formic acid in water and mobile phase B was 0.1% formic acid in acetonitrile. The separation was carried out by a 120 min gradient from 3% to 90% of phase B.

### 3.3. Data Analysis

MS data were analyzed using the MaxQuant (v 1.5.4.1) [36] program against the SwissProt Human database with an initial precursor mass error of 10 ppm. The minimum peptide length for identification was set to 7 amino acids; the match between the runs option was activated. The cutoff false discovery rate (FDR) for proteins and peptides was set to 0.01 (1% FDR). At least two unique peptide identifications per protein were required. Label-free quantitative analysis was performed in order to determine the significantly changed proteins. For label-free quantification, raw mass-spectrometry files were processed by the MaxQuant software using a specific algorithm which included feature detection, first and main search, and peptide/protein identification and quantification. Quantification of peptides recognized on the basis of mass and retention time but identified in other LC-MS/MS runs (“match between the runs” option in MaxQuant). Proteins quantification was carried out using label-free quantification (LFQ) intensities of peptides across all samples and represented by a normalized intensity profile that is generated according to the algorithms described by Cox, J. et al [36]. Hierarchical clustering of proteomic composition was performed using logarithmized LFQ intensities.

To analyze the correlation between samples and technical runs, Pearson’s coefficient was calculated. It shows a good correlation (about 0.9) between sample runs and acceptable correlation for inter-individual variability in groups of samples. Coefficient of variation plotted against the abundance of the proteins demonstrates variations of the plasma proteins in the data set (Figure S1). Additionally, the distribution of coefficients of variation is demonstrated in different time points for significantly changed proteins on +1 days after landing (Figure S2).

A two-sample Welch’s *t*-test with Benjamini–Hochberg correction was applied to identify the significantly changing proteins in the study groups (*p*-value < 0.01). Protein–protein interactions were analyzed using the STRING database (v 11.0). The minimum coefficient of interaction score was 0.4; the PPI enrichment *p*-value was < 1.0 × 10^16^. The interactions included physical and functional associations derived from computational prediction, automated text mining, co-expression databases and genomic context prediction aggregated from other databases. As a result, STRING [37] generated network images using the spring model. Each association had a score that was derived from the *p*-value that indicated the enrichment of similar processes and functions, etc. Only associations with *p* < 0.05 were included in the final networks. Protein categorical annotations were derived from GeneOntology via the SwissProt Human database. All samples were analyzed in triplicate. The Pearson’s coefficients were calculated and showed a good correlation (>0.9) between sample runs. The mass spectrometric proteomic data have been deposited to the ProteomeXchange Consortium via the PRIDE [38] partner repository with the dataset identifier PXD013305.

## Figures and Tables

**Table 1:** The list of proteins significantly changed (*p* < 0.01) on the 21st day of the head-down bed rest (HDBR) ground-based experiment with respect to their background levels (the day before the experiment). Proteins were identified by pair-wise comparison (Welch’s *t*-test). Red/blue symbol—the increase/decrease of the protein level with respect to the background.

Protein IDs	Gene Names	Protein Names	Unique Peptides	*p*-Value	*t*-Test Difference		HDBR 21 Day	Background
Q9Y490	*TLN1*	Talin-1	46	8.4 × 10^−^^6^	−4.6	˅	16.6 ± 0.6	21.2 ± 1.8
P01861	*IGHG4*	Ig gamma-4 chain C region	3	5.2 × 10^−^^7^	−4.2	˅	18.5 ± 0.6	22.6 ± 1.8
P02655	*APOC2*	Apolipoprotein C-II;Proapolipoprotein C-II	5	9.9 × 10^−^^6^	−3.6	˅	19.4 ± 1.2	23.1 ± 1.2
P12814	*ACTN1*	Alpha-actinin-1	24	1.2 × 10^−^^6^	−3.3	˅	16.7 ± 0.5	20 ± 2.3
P01834	*IGKC*	Ig kappa chain C region	5	6.7 × 10^−^^4^	−2.5	˅	19.3 ± 1.6	21.8 ± 3
P63261; P60709	*ACTG1; ACTB*	Actin. cytoplasmic 2; Actin. cytoplasmic 1	6	2.9 × 10^−^^4^	−2.1	˅	18.4 ± 0.6	20.6 ± 2.5
O00187	*MASP2*	Mannan-binding lectin serine protease 2	6	1.5 × 10^−^^5^	−1.9	˅	18.6 ± 1.1	20.6 ± 1.1
O00391	*QSOX1*	Sulfhydryl oxidase 1	13	1.3 × 10^−^^4^	−1.4	˅	18.8 ± 0.6	20.2 ± 1.4
P55058	*PLTP*	Phospholipid transfer protein	10	1.6 × 10^−^^6^	−1.4	˅	18.1 ± 0.4	19.5 ± 0.8
Q99878; Q96KK5; Q9BTM1; Q93077; Q8IUE6; Q7L7L0; P20671; P0C0S8; P04908; P16104	*HIST1H2AJ; HIST1H2AH; H2AFJ; HIST1H2AC; HIST2H2AB; HIST3H2A; HIST1H2AD; HIST1H2AG; HIST1H2AB; H2AFX*	Histone H2A	1	1.2 × 10^−^^5^	−0.9	˅	17.2 ± 0.2	18.2 ± 0.9
P05154	*SERPINA5*	Plasma serine protease inhibitor	9	3.1 × 10^−^^5^	0.7	˄	19.5 ± 0.2	18.8 ± 0.5
P07358	*C8B*	Complement component C8 beta chain	12	9.7 × 10^−^^5^	0.8	˄	20.5 ± 0.5	19.7 ± 1
P03952	*KLKB1*	Plasma kallikrein	9	1.3 × 10^−^^4^	0.9	˄	21.3 ± 0.6	20.4 ± 1
P01024	*C3*	Complement C3	93	5.1 × 10^−^^5^	1.0	˄	27.2 ± 0.5	26.2 ± 1.2
P22792	*CPN2*	Carboxypeptidase N subunit 2	6	6.8 × 10^−^^4^	1.1	˄	22 ± 0.6	21 ± 0.7
P04114	*APOB*	Apolipoprotein B-100	192	3.0 × 10^−^^4^	1.1	˄	26.1 ± 0.3	25.1 ± 1.7
P06681	*C2*	Complement C2	12	2.6 × 10^−^^4^	1.2	˄	21.1 ± 0.4	19.9 ± 1.3
P02766	*TTR*	Transthyretin	10	5.2 × 10^−^^4^	1.2	˄	23.1 ± 0.9	21.9 ± 1.7
P25311	*AZGP1*	Zinc-alpha-2-glycoprotein	13	5.6 × 10^−^^6^	1.4	˄	23 ± 0.6	21.6 ± 1.2
P02774	*GC*	Vitamin D-binding protein	13	8.6 × 10^−^^6^	1.4	˄	23 ± 1	21.6 ± 1.3
P04196	*HRG*	Histidine-rich glycoprotein	10	6.7 × 10^−^^7^	1.4	˄	23.5 ± 0.8	22.1 ± 1
Q06033	*ITIH3*	Inter-alpha-trypsin inhibitor heavy chain H3	10	6.4 × 10^−^^7^	1.4	˄	20.9 ± 0.5	19.5 ± 1.2
P01019	*AGT*	Angiotensinogen	10	2.7 × 10^−^^5^	1.4	˄	22 ± 1.1	20.6 ± 1.3
P08519	*LPA*	Apolipoprotein(a)	7	4.8 × 10^−^^4^	1.5	˄	21.5 ± 1.3	20 ± 0.9
P01023	*A2M*	Alpha-2-macroglobulin	36	2.0 × 10^−^^6^	1.5	˄	25.4 ± 0.6	23.9 ± 1.6
P01042	*KNG1*	Kininogen-1	16	1.1 × 10^−^^7^	1.6	˄	23.1 ± 0.8	21.5 ± 1.1
O75882	*ATRN*	Attractin	10	5.0 × 10^−^^4^	1.6	˄	21.3 ± 0.4	19.7 ± 1.5
P00751	*CFB*	Complement factor B	18	6.8 × 10^−^^8^	1.7	˄	23.2 ± 0.5	21.5 ± 1.2
P35858	*IGFALS*	Insulin-like growth factor-binding protein complex acid labile subunit	11	7.9 × 10^−^^5^	1.7	˄	20.9 ± 0.7	19.2 ± 1.6
P05546	*SERPIND1*	Heparin cofactor 2	14	1.3 × 10^−^^8^	1.7	˄	22.6 ± 0.7	20.9 ± 0.9
P02750	*LRG1*	Leucine-rich alpha-2-glycoprotein	8	1.2 × 10^−^^7^	1.8	˄	22.4 ± 0.4	20.7 ± 1.2
Q16610	*ECM1*	Extracellular matrix protein 1	9	1.7 × 10^−^^4^	1.8	˄	20.7 ± 0.5	18.9 ± 1.7
Q14624	*ITIH4*	Inter-alpha-trypsin inhibitor heavy chain H4	37	3.2 × 10^−^^9^	1.9	˄	25.1 ± 0.4	23.2 ± 1.2
P29622	*SERPINA4*	Kallistatin	14	5.0 × 10^−^^7^	1.9	˄	21.5 ± 0.3	19.5 ± 1.5
P08697	*SERPINF2*	Alpha-2-antiplasmin	16	8.0 × 10^−^^9^	2.0	˄	22.6 ± 0.5	20.5 ± 1.4
P01008	*SERPINC1*	Antithrombin-III	22	9.4 × 10^−^^8^	2.1	˄	24.1 ± 0.8	22 ± 1.6
P04217	*A1BG*	Alpha-1B-glycoprotein	12	1.0 × 10^−^^7^	2.1	˄	23.2 ± 0.9	21.1 ± 1.5
P01009	*SERPINA1*	Alpha-1-antitrypsin	23	4.9 × 10^−^^8^	2.2	˄	24.9 ± 1.2	22.7 ± 1.2
P05155	*SERPING1*	Plasma protease C1 inhibitor	14	7.4 × 10^−^^7^	2.3	˄	24 ± 1.3	21.7 ± 1.8
P01011	*SERPINA3*	Alpha-1-antichymotrypsin	19	1.6 × 10^−^^9^	2.6	˄	24.9 ± 1	22.2 ± 1.3
P02790	*HPX*	Hemopexin	14	4.6 × 10^−^^8^	2.7	˄	25.9 ± 1.3	23.2 ± 1.8
P19827	*ITIH1*	Inter-alpha-trypsin inhibitor heavy chain H1	24	4.1 × 10^−^^9^	2.8	˄	24.6 ± 0.6	21.8 ± 1.9
P01031	*C5*	Complement C5	34	2.7 × 10^−^^9^	3.2	˄	23.6 ± 0.3	20.5 ± 1.9
P19823	*ITIH2*	Inter-alpha-trypsin inhibitor heavy chain H2	32	7.3 × 10^−^^10^	3.2	˄	25.6 ± 0.6	22.4 ± 1.8

**Table 2:** The list of proteins significantly changed (*p* < 0.01) on the 21^st^ day of the dry immersion ground-based experiment with respect to their background levels (the day before the experiment). Proteins were identified by pair-wise comparison (Welch’s *t*-test). Red/blue symbol—the increase/decrease of protein level with respect to the background.

Protein IDs	Gene Names	Protein Names	Unique Peptides	*p*-Value	*t*-Test Difference		Immersion 21 Day	Background
P01024	*C3*	Complement C3	93	6.8 × 10^−^^6^	1.0	˄	27.2 ± 0.5	26.2 ± 1.2
P02766	*TTR*	Transthyretin	10	1.3 × 10^−^^5^	1.1	˄	23 ± 0.4	21.9 ± 1.7
P04114	*APOB*	Apolipoprotein B-100; Apolipoprotein B-48	192	4.7 × 10^−^^7^	1.6	˄	25.4 ± 0.6	24.2 ± 1.5
O75636	*FCN3*	Ficolin-3	10	9.1 × 10^−^^6^	1.7	˄	27.7 ± 1.1	26.4 ± 1.4
P00450	*CP*	Ceruloplasmin	39	5.6 × 10^−^^6^	1.2	˄	23.7 ± 0.7	22.3 ± 1.7
P00734	*F2*	Prothrombin	10	3.1 × 10^−^^5^	1.7	˄	21.5 ± 0.6	20.1 ± 1.4
P00736	*C1R*	Complement C1r subcomponent	14	7.5 × 10^−^^6^	2.4	˄	26.6 ± 0.3	25.1 ± 1.7
P00738	*HP*	Haptoglobin	7	1.8 × 10^−^^5^	1.9	˄	22.1 ± 0.7	20.5 ± 2.1
P01857	*IGHG1*	Ig gamma-1 chain C region	4	2.2 × 10^−^^6^	2.6	˄	22.7 ± 0.4	21 ± 2.1
P01871; P04220	*IGHM*	Ig mu chain C region	11	4.3 × 10^−^^6^	2.0	˄	24.7 ± 0.9	22.9 ± 2.5
P02647	*APOA1*	Apolipoprotein A-I	28	2.2 × 10^−^^5^	1.3	˄	23.4 ± 0.7	21.6 ± 2.2
P02652	*APOA2*	Apolipoprotein A-II	6	1.2 × 10^−^^6^	3.3	˄	25.4 ± 0.9	23.6 ± 2.6
P02671	*FGA*	Fibrinogen alpha chain	48	1.2 × 10^−^^6^	2.9	˄	23.4 ± 0.6	21.6 ± 2.1
P02675	*FGB*	Fibrinogen beta chain	26	1.8 × 10^−^^7^	3.3	˄	23.6 ± 0.9	21.7 ± 2.5
P02679	*FGG*	Fibrinogen gamma chain	28	2.4 × 10^−^^7^	3.1	˄	21.5 ± 0.9	19.6 ± 1.9
P02747	*C1QC*	Complement C1q subcomponent subunit C	5	3.0 × 10^−^^5^	1.3	˄	27.6 ± 0.7	25.6 ± 2.1
P02751	*FN1*	Fibronectin	72	1.3 × 10^−^^7^	3.8	˄	24.9 ± 1	22.9 ± 2.3
P02760	*AMBP*	Protein AMBP	5	1.2 × 10^−^^6^	1.4	˄	24.2 ± 0.9	22.1 ± 2.3
P02787	*TF*	Serotransferrin	21	1.8 × 10^−^^6^	2.2	˄	25.1 ± 0.9	22.9 ± 2.8
P04004	*VTN*	Vitronectin	15	6.5 × 10^−^^6^	2.1	˄	24.3 ± 1.1	22.1 ± 2.1
P06727	*APOA4*	Apolipoprotein A-IV	34	1.3 × 10^−^^5^	1.8	˄	24.2 ± 1.2	22 ± 2.6
P07225	*PROS1*	Vitamin K-dependent protein S	14	1.9 × 10^−^^6^	1.9	˄	22.4 ± 0.6	20.2 ± 2.4
P08603	*CFH*	Complement factor H	18	2.0 × 10^−^^6^	2.1	˄	23.6 ± 1	21.2 ± 2.9
P09871	*C1S*	Complement C1s subcomponent	17	4.5 × 10^−^^7^	2.6	˄	24.7 ± 0.8	22 ± 2.7
P0C0L5	*C4B*	Complement C4-B	5	7.1 × 10^−^^7^	2.0	˄	25 ± 1.4	22.3 ± 2.6
P10909	*CLU*	Clusterin	17	9.1 × 10^−^^7^	3.1	˄	28 ± 1.2	25.1 ± 3.1
P12259	*F5*	Coagulation factor V	39	1.4 × 10^−^^5^	1.6	˄	27.8 ± 1.2	24.6 ± 2.7
P22352	*GPX3*	Glutathione peroxidase 3	6	2.3 × 10^−^^6^	1.9	˄	25.8 ± 1.1	22.7 ± 3.4
P27169	*PON1*	Serum paraoxonase/arylesterase 1	10	5.0 × 10^−^^6^	2.2	˄	24.9 ± 1.4	21.6 ± 3.2
P80108	*GPLD1*	Phosphatidylinositol-glycan-specific phospholipase D	25	1.5 × 10^−^^5^	1.8	˄	28.2 ± 1	24.9 ± 3.1
Q96KN2	*CNDP1*	Beta-Ala-His dipeptidase	18	1.7 × 10^−^^6^	2.2	˄	26.7 ± 0.5	22.9 ± 3.7

**Table 3:** The list of proteins significantly changed (*p* < 0.01) on +1 day after long-term spaceflight. Proteins were identified by pair-wise comparison (Welch’s *t*-test). Red/blue symbol—the increase/decrease of protein level with respect to the background.

Protein IDs	Gene Names	Protein Names	Unique Peptides	*p*-Value	*t*-Test Difference		Log2 (LFQ Intensity) Background	Log2 (LFQ Intensity) + 1 Day	Log2 (LFQ Intensity) + 7 Days
P02787	*TF*	Serotransferrin	26	1.70 × 10^−^^7^	2.5	˄	20.7 ± 2.1	23.2 ± 1.4	21.5 ± 1.9
P04217	*A1BG*	Alpha-1B-glycoprotein	11	3.30 × 10^−^^5^	2.2	˄	17.1 ± 0.8	19.3 ± 1.1	18.3 ± 1.9
P01008	*SERPINC1*	Antithrombin-III	20	3.60 × 10^−^^8^	1.9	˄	19.1 ± 0.9	20.9 ± 1.2	19.6 ± 1.6
P0DJI8	*SAA1*	Serum amyloid A-1 protein	4	8.40 × 10^−^^5^	1.8	˄	19.6 ± 1.1	21.4 ± 1.4	20.2 ± 0.8
P05155	*SERPING1*	Plasma protease C1 inhibitor	11	1.70 × 10^−^^4^	1.8	˄	17.9 ± 1	19.7 ± 1.4	19.1 ± 1.4
P00751	*CFB*	Complement factor B	13	9.50 × 10^−^^6^	1.8	˄	18.1 ± 1.3	19.9 ± 1.2	18.7 ± 1.5
P01011	*SERPINA3*	Alpha-1-antichymotrypsin	20	1.20 × 10^−^^6^	1.7	˄	19.1 ± 1.1	20.7 ± 1.1	20.3 ± 0.9
P01023	*A2M*	Alpha-2-macroglobulin	40	5.60 × 10^−^^5^	1.4	˄	21.3 ± 1.8	22.8 ± 1.4	22.3 ± 1
P01009	*SERPINA1*	Alpha-1-antitrypsin	25	2.30 × 10^−^^6^	1.1	˄	21.2 ± 0.9	22.4 ± 1.1	21.9 ± 0.8
P00738	*HP*	Haptoglobin	7	2.50 × 10^−^^4^	1	˄	21.1 ± 0.7	22.2 ± 1.4	21.3 ± 1.1
P21333	*FLNA*	Filamin-A	11	2.20 × 10^−^^4^	−0.6	˅	19.5 ± 0.2	18.9 ± 0.2	19.1 ± 0
Q15166	*PON3*	Serum paraoxonase/lactonase 3	7	6.80 × 10^−^^4^	−0.6	˅	19.6 ± 0.4	19 ± 0.7	19.4 ± 0.6
O00391	*QSOX1*	Sulfhydryl oxidase 1	16	1.50 × 10^−^^4^	−0.7	˅	20.5 ± 0.6	19.8 ± 0.8	20.3 ± 0.6
P12259	*F5*	Coagulation factor V	26	6.30 × 10^−^^4^	−0.7	˅	21.1 ± 0.9	20.4 ± 0.8	20.9 ± 1
P05160	*F13B*	Coagulation factor XIII B chain	8	3.30 × 10^−^^4^	−0.8	˅	20.4 ± 0.9	19.6 ± 0.7	20 ± 0.9
P33151	*CDH5*	Cadherin-5	8	4.70 × 10^−^^4^	−0.8	˅	20 ± 0.4	19.2 ± 0.9	19.4 ± 0.7
P00488	*F13A1*	Coagulation factor XIII A chain	26	3.70 × 10^−^^5^	−0.8	˅	21.3 ± 0.8	20.5 ± 0.8	21 ± 0.9
P12830	*CDH1*	Cadherin-1	7	2.10 × 10^−^^4^	−0.8	˅	18.8 ± 0.6	18 ± 0.4	18.8 ± 0.4
Q12805	*EFEMP1*	EGF-containing fibulin-like extracellular matrix protein 1	5	4.40 × 10^−^^6^	−1.2	˅	19.4 ± 0.5	18.1 ± 0.7	19 ± 0.7

## Supplementary Materials

Supplementary materials can be found at https://www.mdpi.com/1422-0067/20/13/3194/s1. All LC-MS/MS and proteomic data are available via ProteomeXchange with identifier PXD013305.
