# Supplementary material for: The Effects of Spaceflight Factors on the Human Plasma Proteome, Including Both Real Space Missions and Ground-Based Experiments
Source: Int J Mol Sci. 2019 Jun 29;20(13):3194. doi: 10.3390/ijms20133194 (PMC6651200; doi:10.3390/ijms20133194)
Supplement: Supplementary file 1 [file ijms-20-03194-s001.pdf]

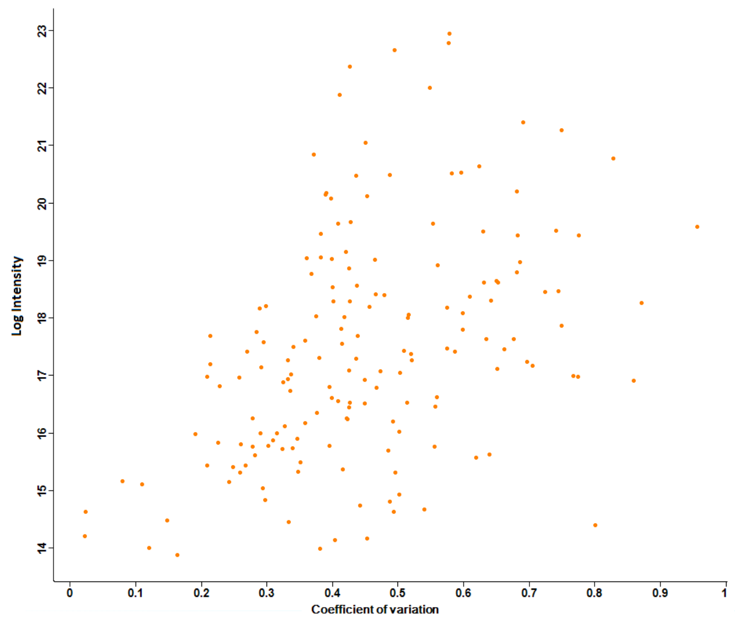

Figure 1S. Coefficient of variation plotted against the abundance of the proteins demonstrates variations of the plasma proteins in the data set .

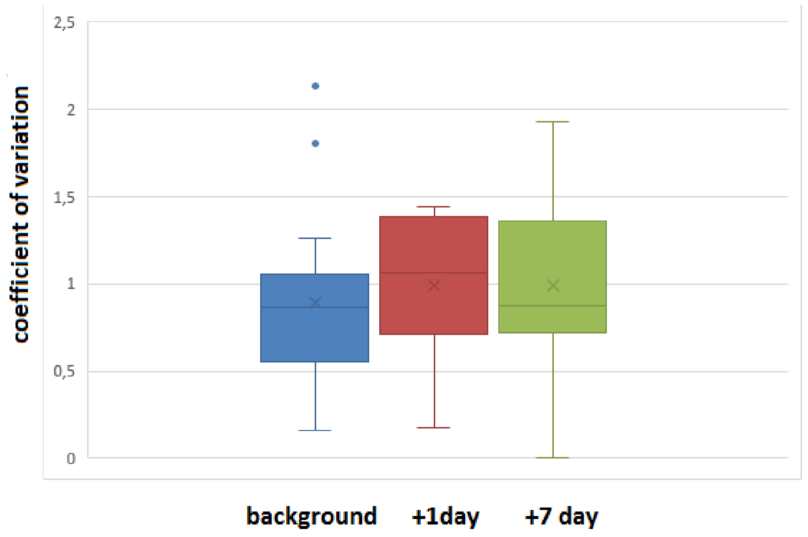

Figure 2S. Distribution of coefficients of variation in different time points for significantly changed proteins on +1 day after landing.
